# Association between morphine exposure and impaired brain development on term-equivalent age brain magnetic resonance imaging in very preterm infants

**DOI:** 10.1038/s41598-022-08677-0

**Published:** 2022-03-16

**Authors:** Mountasser M. Al-Mouqdad, Dima Z. Jamjoom, Roya Huseynova, Thanaa M. Khalil, Yasmeen S. Asfour, Bushra A. Albeshri, Nadia A. Basodan, Fuddah Assiri, Suzan S. Asfour

**Affiliations:** 1 Neonatal Intensive Care, Hospital of Paediatrics, King Saud Medical City, Al Imam Abdul Aziz Ibn Muhammad Ibn Saud, Riyadh, 12746 Saudi Arabia; 2 Radiology and Medical Imaging Department, College of Medicine, King Saud University, Riyadh, Saudi Arabia; 3 Obstetric and Gynecology Department, Maternity Hospital, King Saud Medical City, Riyadh, Saudi Arabia; 4 Obstetric and Gynecology Department, Family Care Hospital, Riyadh, Saudi Arabia; 5 Pharmacy Department, Pharmaceutical Care Services, King Saud Medical City, Riyadh, Saudi Arabia; 6 Clinical Pharmacy Department, Pharmaceutical Care Services, King Saud Medical City, Riyadh, Saudi Arabia

**Keywords:** Medical research, Neurology

## Abstract

To investigate the relationship between morphine exposure in the first week of life and brain injury on term-equivalent age magnetic resonance imaging (MRI) in very preterm infants. A retrospective study included 106 infants with a birth weight of < 1500 g who were born at King Saud Medical City at ≤ 32 gestational weeks, were admitted to the neonatal intensive care unit, and underwent term-equivalent age or pre-discharge brain MRI. A univariate analysis in addition to modified log-Poisson regression with a robust variance estimator was applied, and the effect of early morphine exposure and cumulative dose in the first seven days on brain morphology and growth at term-equivalent age was determined using the Kidokoro score. Sixty-eight (64.2%) infants had received morphine in the first week of life (median cumulative dose: 1.68 mg/kg, interquartile range 0.48–2.52 mg/kg). Early initiation of morphine administration was significantly associated with high total white matter (adjusted relative risk [aRR] 1.32, 95% confidence interval [CI] 1.01–1.72) and cerebellum (aRR 1.36, 95% CI 1.03–1.81) scores and a small cerebellar volume (aRR 1.28, 95% CI 1.02–1.61). Morphine exposure in the first week of life was independently associated with white matter and cerebellar injury on term-equivalent age brain MRI in very preterm infants.

## Introduction

Preterm infants frequently undergo painful procedures during their neonatal intensive care unit (NICU) stay. It is well established that preterm infants can feel pain^1^ and that pain worsens neonatal outcomes, has long-term effects on developmental milestones, and leads to changes in brain morphology^2,3^. Therefore, neonatal pain is a major concern, and neonatologists must strive to minimize it.

Morphine, an opioid analgesic, is commonly used in the NICU to reduce neonatal pain^4,5^. However, its effects on short- and long-term outcomes are unclear. Early randomized trials that investigated short-term neonatal outcomes following morphine exposure obtained controversial conclusions. While the Neonatal Outcome and Prolonged Analgesia in Neonates trial, which had a small sample, found that infants who received morphine had low rates of poor neurological outcomes^6^, the Neurologic Outcomes and Pre-emptive Analgesia in Neonates (NEOPAIN) trial, which had a large sample, showed that infants who received open-label morphine use had high rates of poor neurological outcomes, including severe intraventricular hemorrhage (IVH) or periventricular leukomalacia (PVL), and neonatal death^7^. In recent studies, we found that morphine exposure within the first few days of life was associated with an increased incidence of IVH and brain functional depression on EEG/aEEG, and death in preterm infants with a gestational age of < 28 weeks^8,9^.

Previous studies on the effect of morphine on brain volume at term-equivalent age reported conflicting results. Steinhorn et al. found that low doses of morphine were not associated with changes in brain volume at term-equivalent age^10^; however, the participants of their study showed dysregulated behavior despite having normal cognitive and motor development at the age of 2 years and no significant psychoneurological impairment at the age of 7 years. Another study found that early morphine exposure was significantly correlated with decreased cerebellar volume and poor motor and cognitive outcomes at the age of 18 months^11,12^. In contrast, the European morphine trial found no impact of morphine on severe IVH, PVL or death; when the participants were followed up, it was found that the morphine group had a lower intelligence quotient at the age of 5 years and better executive function at the age of 8–9 years than the control group^13–15^. Morphine exposure in the neonatal period has also been associated with decreased brain volume at the age of 10 years^16^.

Different studies have used different scales to estimate the severity of brain injury on magnetic resonance imaging (MRI) at term-equivalent age, leading to inconsistent outcomes^17–19^. Therefore, the influence of morphine exposure on brain morphology and growth at term-equivalent age should be studied in various populations using a comprehensive and objective MRI scoring method^20^. Recent studies have reported that the Kidokoro score is highly reliable for detecting brain abnormalities and predicting motor, learning, and memory performance in children aged 2 and 7 years^21–23^. It is a standardized scoring system identifying the global and regional alterations of brain structure by measuring precise quantitative biometrics. Moreover, it is categorizing the injury of cerebral white matter, cortical gray matter, deep gray matter, and cerebellum in definite score that can help to recognize the severity of brain injury.

Therefore, this study aimed to investigate the effects of morphine exposure in the first week of life on brain morphology and growth at term-equivalent age in preterm infants using the Kidokoro score.

## Results

**Figure 1:**
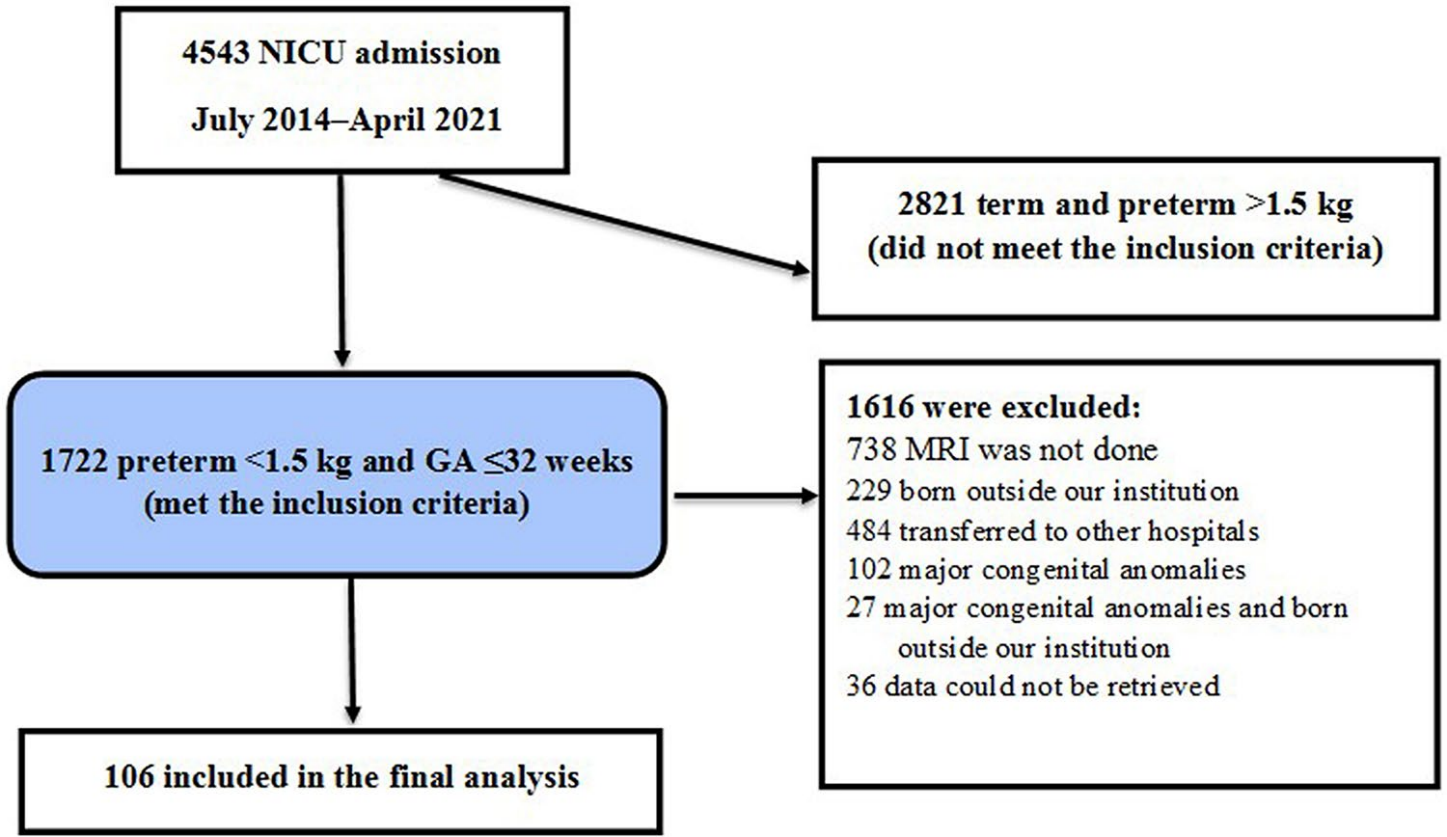
Flow chart of patient selection. *GA* gestational age, *NICU* neonatal intensive care unit.

Of 1722 preterm infants with a gestational age ≤ 32 weeks and birth weight < 1500 g admitted to the NICU (level 3) during the study period, 106 met the selection criteria and were eligible for inclusion in the final analysis (Fig. 1).

Sixty-eight (64.2%) infants received morphine infusion in the first week of life, with a median cumulative dose of 1.68 mg/kg (IQR 0.48–2.52 mg/kg). The demographic characteristics of the mothers and infants stratified according to morphine exposure are presented in Table 1. Infants with morphine exposure in the first week of life had a significantly lower birth weight and gestational age than those without morphine exposure in the first week of life (P < 0.001, P = 0.003, respectively).

**Table 1:** Demographic and clinical characteristics of the mothers and premature infants by morphine exposure in the first 7 days of life (n = 106).

Parameters	Exposure to morphine in the first 7 days of life		(P value)
	No (n = 38)	Yes (n = 68)	
Gestational age, weeks	30 (28–32)	27 (26–29)	< 0.001*
Birth weight, grams	1130 (977.5–1302.5)	955 (806.25–1200)	0.003*
Booked	3 (7.9)	8 (11.8)	0.74
Antenatal steroid	18 (47.4)	24 (35.3)	0.3
Cesarean section	20 (52.6)	39 (57.4)	0.69
Maternal hypertension	14 (36.8)	10 (14.7)	0.01*
Gestational diabetes	1 (2.6)	4 (5.9)	0.65
Chorioamnionitis	1 (4)	0 (0)	0.35
1-min Apgar score	5 (3–7)	4 (3–6)	0.06
5-min Apgar score	7 (6–8)	7 (6–7)	0.05
RDS surfactant use	18 (47.4)	36 (92.6)	< 0.001*
Male sex	20 (52.6)	42 (51.8)	0.41
PDA treatment	4 (10.5)	11 (16.2)	0.56
NEC (surgical)	0 (0)	7 (10.3)	0.05
IVH	14 (36.8)	48 (70.6)	0.001*
Pneumothorax	1 (14.3)	6 (85.7)	0.42
Pulmonary hemorrhage	2 (5.3)	12 (17.6)	0.08
Inotropes	8 (21.1)	45 (66.2)	< 0.001*
Hydrocortisone	1 (2.6)	14 (20.6)	0.01*
UAC	10 (26.3)	19 (27.9)	> 0.99
UVC	27 (71.1)	56 (82.4)	0.22
Total noninvasive ventilation (days)	15 (6.75–25.5)	28 (17.13–46.25)	< 0.001*
Total invasive ventilation (days)	3 (0–10.5)	17.5 (9.25–39.75)	< 0.001*
Total TPN days	20 (6.75–49.6250)	29 (20–78.5)	0.06
LOHS	56.5 (44–79.25)	94 (67–126.5)	< 0.001*
Age at MRI	37 (35–38.25)	38 (35–41)	0.09
LOS	9 (23.7)	34 (50)	0.013*
Postnatal steroid	8 (21.1)	21 (30.8)	0.05

Data are presented as median (IQR), or number (%), as appropriate.

Significant values are in bold.

*RDS* Respiratory distress syndrome, *PDA* Patent ductus arteriosus, *NEC* necrotizing enterocolitis, *IVH* Intraventricular hemorrhage, *UAC* umbilical arterial catheter, *UVC* umbilical venous catheter, *TPN* Total parenteral nutrition, *LOHS* Length of hospital stay, *MRI* magnetic resonance imaging, *LOS* Late onset sepsis.

*Statistically significant at 5% level.

Infants with early morphine exposure received more surfactant, required more inotropes and hydrocortisone, and were more likely to require mechanical ventilation than those without early morphine exposure (P < 0.001, P < 0.001, P = 0.01, and P < 0.001, respectively). Furthermore, they had higher rates of IVH and late-onset sepsis (P = 0.001 and P = 0.01, respectively) and lower survival rates than infants without early morphine exposure (P < 0.001; Table 1).

Univariate analysis revealed that early morphine exposure was significantly associated with poor neurological outcomes (Table 2). In addition, cumulative morphine exposure was significantly associated with focal signal abnormality, myelination delay, CC thinning, lateral ventricular dilatation, total WM score, volume reduction in deep GM score, total deep GM score, cerebellum signal abnormality, cerebellar volume reduction score, and total cerebellar score (P = 0.01, P = 0.03, P < 0.001, P < 0.001, P < 0.001, P < 0.001, P = 0.007, P < 0.001, P < 0.001, and P < 0.001, respectively). On the other hand, early morphine exposure was not associated with cystic lesions, WM volume reduction, signal abnormality in cortical GM score, delayed gyral maturation, increased extracerebral space, total cortical GM score, and deep GM signal abnormalities. Multivariable regression analysis revealed that early morphine exposure remained significantly associated with increased total WM score after adjusting for variables that were found to be significant in the univariate analysis (adjusted relative risk [aRR] 1.32, 95% confidence interval [CI] 1.01–1.72). Additionally, early morphine exposure was associated with cerebellar volume reduction (aRR 1.28, 95% CI 1.02–1.61) and increased total cerebellum score (aRR 1.36, 95% CI 1.03–1.81; Table 3). Besides, in the multivariable regression analysis we found that cumulative morphine doses were significantly associated with myelination delay (aRR 1.11, 95% CI 1.03–1.20), thinning of the corpus callosum (aRR 1.42, 95% CI 1.17–1.72), dilated lateral ventricles (aRR 1.20, 95% CI 1.004–1.44), high total white matter score (aRR 1.12, 95% CI 1.03–1.22) as well as high total cerebellar score (aRR 1.14, 95% CI 1.03–1.26).

**Table 2:** Univariate analysis of global brain abnormalities score on TEA-MRI in relation to morphine exposure in first week of life (n = 106).

	Exposure to morphine in the first 7 days of life		P value
	No (38)	Yes (68)	
**White matter score**			
Cystic lesions	0 (0–3)	0 (0–3)	0.13
Focal signal abnormality	0 (0–0.25)	0 (0–2)	0.02*
Myelination delay	1 (1–1)	1 (1–2)	0.06
Thinning of the corpus callosum	0 (0–0.25)	0 (0–2)	0.009*
Dilated lateral ventricles	0 (0–1)	2 (0–3)	0.004*
Volume reduction	1.5 (1–2)	2 (1–2)	0.14
Total white matter score	3 (2–8)	7 (4–11)	< 0.001*
**Cortical gray matter score**			
Signal abnormality	0 (0–0)	0 (0–0)	0.38
Gyral maturation	0 (0–0)	0 (0–0)	0.71
Increased extracerebral space	0 (0–0)	0 (0–0)	0.09
Total cortical gray matter score	0 (0–0.25)	0 (0–1)	0.59
**Deep gray matter score**			
Signal abnormality	0 (0–0)	0 (0–1)	0.06
Volume reduction	0 (0–0)	0 (0–2)	0.01*
Total deep gray matter score	0 (0–0.25)	0 (0–3)	0.01*
**Cerebellum score**			
Signal abnormality	0 (0–0)	0.5 (0–3)	0.001*
Volume reduction	1 (1–2)	3 (1–3)	< 0.001*
Total cerebellar score	1 (1–3)	3 (2–5)	< 0.001*
Global brain abnormality score	5.5 (3.75–14)	11.5 (8–19)	< 0.001*

Data are presented as median (IQR), or number (%), as appropriate.

Significant values are in bold.

*Statistically significant at 5% level.

**Table 3:** Multivariable regression of global brain abnormalities score on TEA-MRI in relation to morphine exposure in first week of life (n = 106).

Outcome	aRR	95% CI	P value
**White matter score**			
Focal signal abnormality	0.68	0.31–1.49	0.33
Thinning of the corpus callosum	1.1	0.52–2.31	0.79
Dilated lateral ventricles	1.01	0.6–1.71	0.95
Total white matter score^a^	1.32	1.01–1.72	0.03*
**Deep gray matter score**			
Volume reduction	0.91	0.42–1.94	0.79
Total deep gray matter score	1.11	0.48–2.58	0.8
**Cerebellum score**			
Signal abnormality	1.19	0.51–2.79	0.67
Volume reduction^b^	1.28	1.02–1.61	0.02*
Total cerebellar score^c^	1.36	1.03–1.81	0.02*
Global brain abnormality score	1.31	0.99–1.74	0.06

Significant values are in bold.

*aRR* adjusted relative risk, *CI* Confidence interval.

*Statistically significant at 5% level.

^a^aRR were adjusted for IVH, inotropes, and total invasive ventilator days.

^b^aRR were adjusted for gestational age, birth weight, IVH, inotropes, hydrocortisone, age at MRI, pulmonary hemorrhage, total invasive ventilator days and PDA.

^c^aRR were adjusted for gestational age, birth weight, antenatal steroid treatment, IVH, inotropes, hydrocortisone, age at MRI, pulmonary hemorrhage, total invasive ventilator days and PDA.

On performing 1:1 matching of infants with and without morphine exposure, [each group was matched for gestational age (1 week)], 34 infants who received morphine were matched with 34 infants who did not receive morphine (Table 4). We found that early morphine exposure remained significantly associated with delayed myelination, CC thinning, and lateral ventricular dilatation in the WM (P = 0.02, P = 0.002, and P = 0.03, respectively). Additionally, morphine exposure in the first week of life was significantly associated with signal abnormality, deep GM volume reduction, and total deep GM score, (P = 0.01, P = 0.008, and P = 0.01, respectively). Furthermore, morphine exposure in the first week of life was significantly associated with cerebellar signal abnormalities and high cerebellar volume reduction, total cerebellar, and global brain abnormality scores (P < 0.001, P = 0.001, P < 0.001, and P = 0.001, respectively; Table 4).

**Table 4:** Univariate analysis of global brain abnormalities score on TEA-MRI in relation to morphine exposure in first week of life after matching (n = 68).

Parameters	Exposure to morphine in the first 7 days of life		P value
	No (34)	Yes (34)	
GA	30 (28–32)	29 (27–31)	0.26
BW	1170 (965–1317.5)	1080 (922.5–1285)	0.21
**White matter score**			
Cystic lesions	0 (0–3)	0.5 (0–4)	0.13
Focal signal abnormality	0 (0–0.25)	0 (0–2)	0.06
Myelination delay	1 (1–1)	1 (1–2)	0.02*
Thinning of the corpus callosum	0 (0–0.25)	1.5 (0–2)	0.002*
Dilated lateral ventricles	0 (0–1)	1.5 (0–3)	0.03*
Volume reduction	1 (1–2)	2 (1–2.25)	0.27
Total white matter score	3 (2–8)	7.5 (4–12.25)	0.003*
**Cortical gray matter score**			
Signal abnormality	0 (0–0)	0 (0–0)	0.89
Gray maturation	0 (0–0)	0 (0–0)	0.3
Increased extracerebral space	0 (0–0)	0 (0–0)	0.16
Total cortical deep gray score	0 (0–0.25)	0 (0–1.25)	0.23
**Deep gray matter score**			
Signal abnormality	0 (0–0)	0 (0–1)	0.01*
Volume reduction	0 (0–0)	0.5 (0–3)	0.008*
Total deep gray score	0 (0–0.25)	0.5 (0–4)	0.01*
**Cerebellum score**			
Signal abnormality	0 (0–0)	1 (0–4)	< 0.001*
Volume reduction	1 (1–2)	2 (1–3)	0.001*
Total cerebellar score	1 (1–2.25)	3 (2–7)	< 0.001*
Global brain abnormality score	5 (3–14)	14.5 (6.75–22)	0.001*

Data are presented as median (IQR), or number (%), as appropriate.

Significant values are in bold.

*GA* gestational age, *BW* birth weight.

*Statistically significant at 5% level.

## Discussion

This study found that preterm infants who had received morphine infusion in the first week of life had higher rates of WM and cerebellar injury than those who had not, even after adjusting for important confounding factors. Furthermore, the severity of injury increased with the cumulative dose of morphine in the first week of life.

Our findings are consistent with those of Zwicker et al., who found that morphine exposure was associated with decreased cerebellar volume at term-equivalent age^11^. Animal studies have also reported similar findings; they found that morphine directly influences cerebellar development by reducing the growth and differentiation of cerebellar cells, decreasing Purkinje cell diameter, reducing molecular layer thickness, and inhibiting neuroblast proliferation^24–26^. Interestingly, these studies showed that the first week of life is a critical period during which morphine exposure has the highest effect. Consequently, we can conclude that cerebellar development in preterm infants can be affected by morphine application as early stage of life.

**Figure 2:**
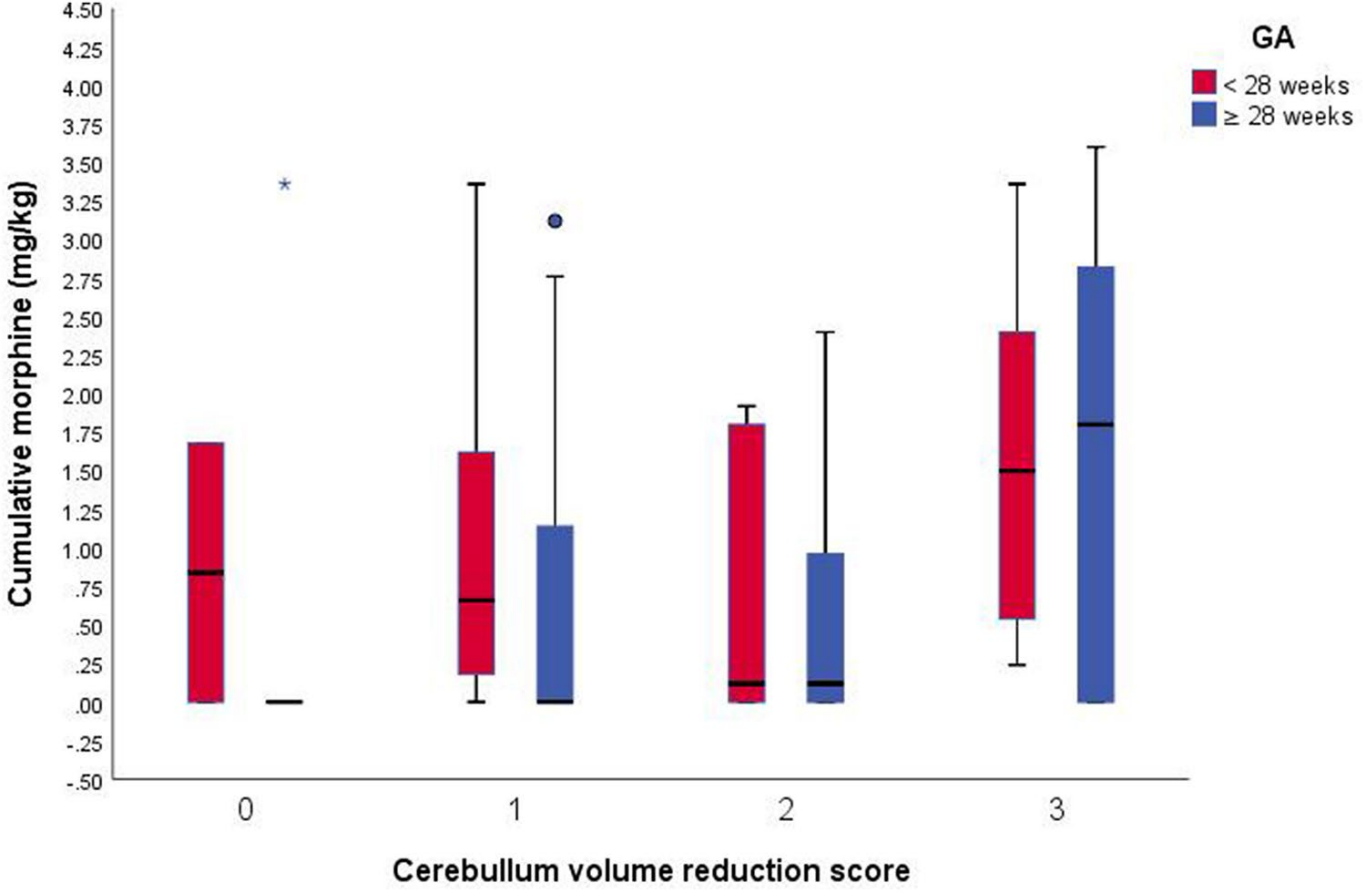
Cerebellar volume score by morphine exposure, adjusted for age at the time of magnetic resonance imaging and stratified by gestational age (GA) at birth (red: < 28 weeks, blue: ≥ 28 weeks). *GA* Gestational age.

**Figure 3:**
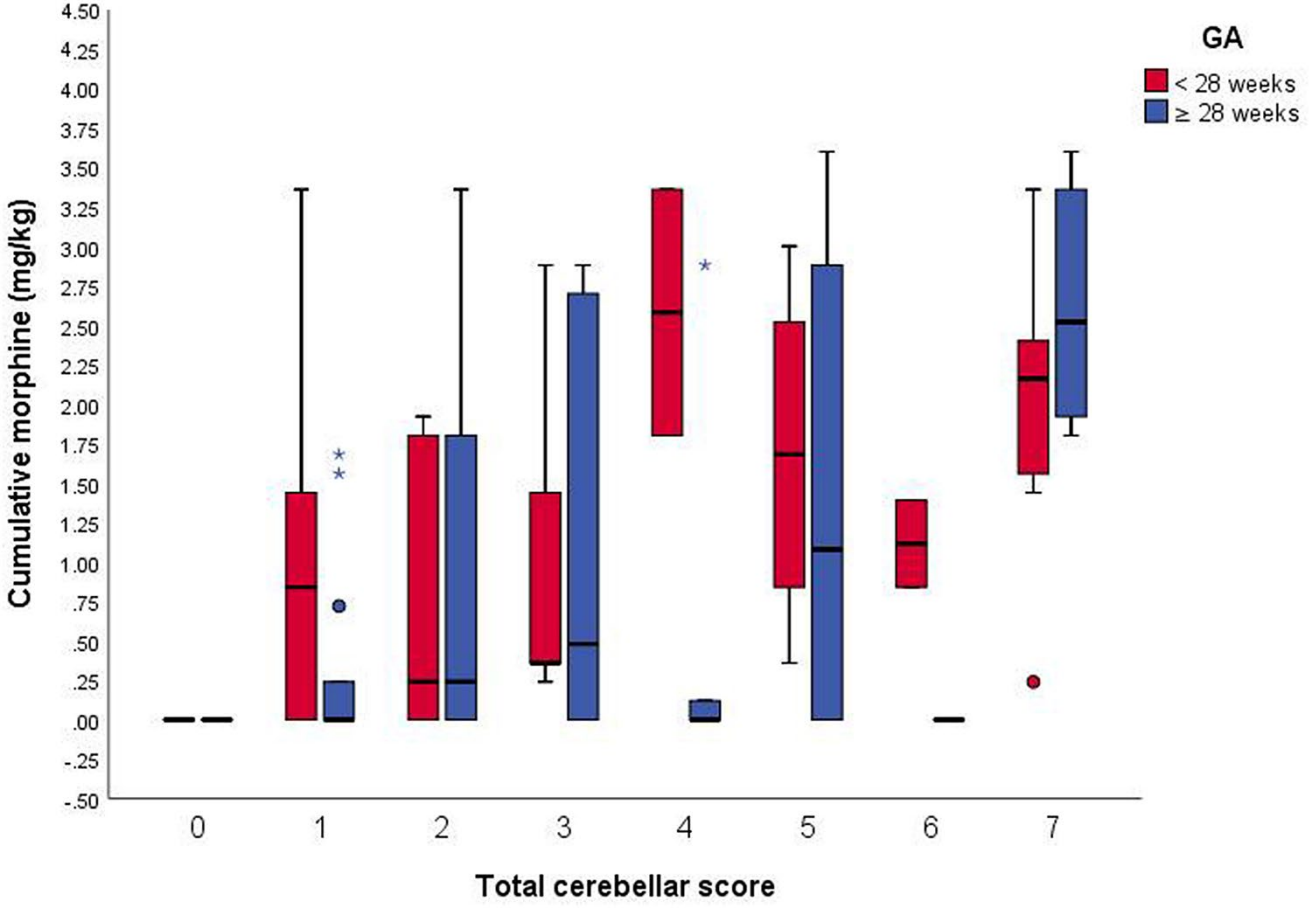
Total cerebellar score by morphine exposure, adjusted for age at the time of magnetic resonance imaging and stratified by gestational age (GA) at birth (red: < 28 weeks, blue: ≥ 28 weeks). *GA* Gestational age.

On classification the preterm infants according to gestational age, we found that in infants who are less than 28 weeks, the severity of cerebellar volume reduction and the total cerebellar score is independent of the cumulative morphine dose. While those who are equal or more than 28 weeks, their score is proportionally relative to morphine cumulative dose (Figs. 2 and 3). The median dose in the study by Zwicker et al. was 1.9 mg/kg, whereas it was 0–1 mg/kg in the study by Steinhorn et al. This suggests that, unlike high cumulative doses, very low cumulative doses of morphine may not affect brain morphology of premature infants who are more than 28 weeks gestation at term-equivalent age. Furthermore, at 20–30 weeks of gestation, the external cortical surface rapidly expands, external granular layer reaches its peak thickness, and Purkinje cells differentiate and secrete Sonic hedgehog, which stimulates the proliferation of granular precursor cells^26,27^. Thus, infants with a gestational age of < 28 weeks demonstrated higher cerebellar injury scores regardless the morphine cumulative dose.

**Figure 4:**
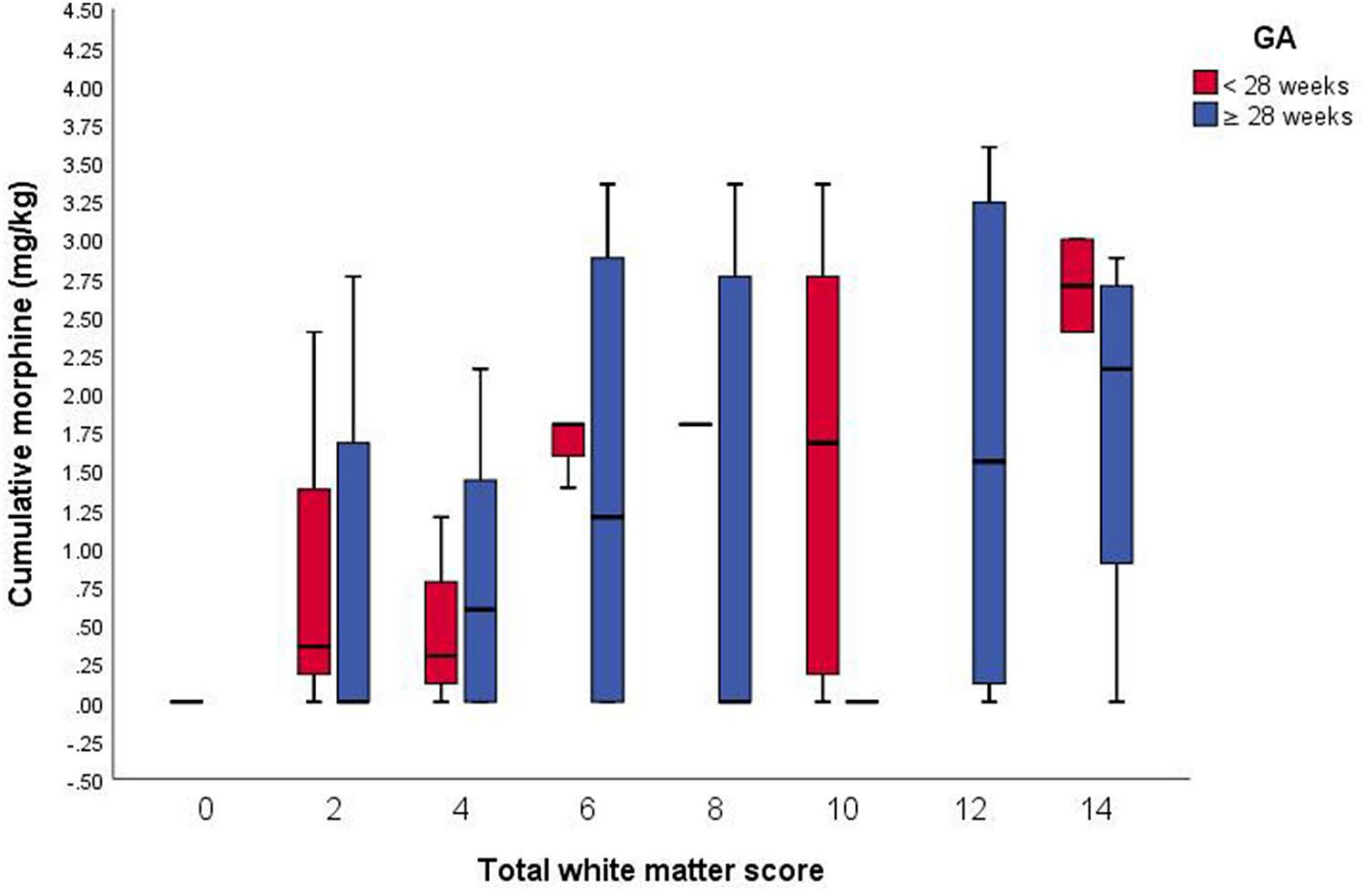
Total white matter score by morphine exposure, adjusted for age at the time of magnetic resonance imaging and stratified by gestational age (GA) at birth (red: < 28 weeks, blue: ≥ 28 weeks). *GA* Gestational age.

Another significant finding of our study was that morphine exposure in the first week of life was associated with a high incidence of white matter injury (WMI) and that the severity of this injury increased with the cumulative dose of morphine. Moreover, infants born at < 28 weeks of gestation are more vulnerable to WMI due to morphine exposure than those born at 28–32 weeks of gestation (Fig. 4). The Kidokoro score not only considers the type of WMI (cystic vs. non-cystic), but also considers CC thickening, myelination, and the status of the lateral ventricles. In general, WMI is the most commonly occurring brain lesion in preterm infants^28^. Although brain ultrasound is very useful for detecting cystic WMI at the bedside, brain MRI is the gold-standard radiologic tool for identifying and grading WMI in preterm infants^19,29^. A recent systematic review demonstrated that the prevalence of WMI on brain MRI is higher in preterm infants born at < 28 weeks of gestation than in those born at ≥ 28 weeks of gestation^30^. Our results are consistent with those of the NEOPAIN trial, which showed that pre-emptive morphine infusion in preterm infants on mechanical ventilation did not increase the rates of severe IVH and PVL on brain ultrasound; however, they found that open-label morphine use was associated with severe IVH, PVL, and/or neonatal death^6^. However, Zwicker et al. and Steinhorn et al. found that morphine exposure was not associated with WMI. This inconsistency in results could be attributed to differences in the genetic and environmental characteristics of the infants with morphine exposure. A recent study assessed the developmental outcomes and behavioral problems at the age of 18 months of approximately 200 preterm infants born at ≤ 32 weeks of gestation who were exposed to morphine during the critical period of brain development. They found that some children developed behavioral problems despite having no history of morphine exposure, indicating that there may be specific genotypes associated with an increased likelihood of having depression and anxiety. Therefore, the internalizing and externalizing behaviors of preterm infants are highly associated with genetic variations in morphine metabolism^31^.

Hypoxemia is another possible mechanism by which morphine exposure can affect the central nervous system. The NEOPAIN trial found that morphine administration does not lead to improved respiratory outcomes in intubated preterm infants, and frequent doses of morphine can result in a prolonged duration of ventilation and oxygen therapy and an increased risk of air leak^32^. Another randomized controlled trial assessed the duration of hypoxemia and fluctuation of vital signs in preterm infants who received morphine before elective intubation. They found that the duration of hypoxemia was prolonged in preterm infants who received morphine before intubation. Infants with morphine exposure-induced hypoxemia may require prolonged intubation and mechanical ventilation, resulting in a delay in achieving full enteral feeding^33^.

American Academy of Pediatrics and Canadian Pediatric Society have stated that the role of continuous sedation/analgesia in the treatment of chronic discomfort is unclear, and the routine use of continuous morphine infusion is not indicated for short durations of mechanical ventilation; it should mainly be reserved for analgesia post major surgery^34^.

The limitations of our study are as follows: First, it is a retrospective observational study, and the decision to start morphine infusion was made by the neonatologist based on their clinical experience. Second, although we adjusted for most of the important confounders, we could not estimate the severity of procedure-induced neonatal pain. Finally, the sample size is relatively small because we excluded infants with clinical factors that might have affected the results.

The strengths of our study are as follows: First, we used a comprehensive global score; this enabled us to evaluate the effect of morphine exposure on most of the regions of the brain. Second, our results did not change even after the groups were matched according to gestational age and birth weight. Finally, to the best of our knowledge, the study population was novel, and similar studies using MRI have not been conducted in our region. Thus, we could highlight the role of genetic and environmental features on the impact of morphine exposure.

In conclusion, morphine exposure during the first week of life is independently associated with high cerebellar and WMI scores on term-equivalent age MRI in preterm infants. Neurodevelopmental follow-up studies in this population are required to elucidate the long-term sequelae of morphine administration during the critical period of brain development.

## Methods

### Study design

This retrospective chart review included a cohort of preterm infants who were admitted to the NICU at King Saud Medical City (KSMC) tertiary referral center between July 2014 and April 2021.

Including level 3, the NICU at KSMC has an average annual admission of 1100 patients. This study was conducted in accordance with the Declaration of Helsinki and Good Pharmacoepidemiology Practice guidelines and was approved by the medical ethical review committee of KSMC (reference number H1RI-25-Feb19-01). The requirement for consent was waived.

### Inclusion and exclusion criteria

We included infants who were born at KSMC at ≤ 32 weeks of gestation, had a birth weight of < 1500 g, were admitted to the NICU. All infants who received morphine infusion in the first week of life were intubated and had undergone brain MRI at term-equivalent age or before discharge. We excluded infants with major congenital anomalies or congenital infection, those who were not born at KSMC, and those whose data could not be retrieved.

### Data collection and follow-up

The infants’ charts from NICU admission until discharge or death were reviewed. Demographic, clinical, and outcome data were obtained. Maternal data, including gestational diabetes mellitus, maternal hypertension, antenatal steroid treatment, and mode of delivery, were also retrieved.

### Study outcome

The primary outcome of this study was brain injury at term-equivalent age as assessed using the Kidokoro score.

### Morphine exposure

Cumulative morphine exposure was calculated from the average daily dose of intravenous morphine in the first week of life and adjusted for daily weight. We sub-classified according to the gestational age, less than 28 and more than or equal 28 gestational age. Then, we correlated the cumulative dose of morphine infusion with total white matter, total cerebellum, and cerebellum volume reduction score.

### Term-equivalent MRI

All included infants underwent MRI without sedation. MRI was performed using a GE Optima MR450w 1.5-T, 70-cm (General Electric, Connecticut, USA) scanner. Three-dimensional spin-echo T1-weighted images, axial and coronal T2-weighted images, axial fluid-attenuated inversion recovery images, and diffusion- and susceptibility-weighted images were obtained.

The images were evaluated using a standardized scoring system developed by Kidokoro et al.^20^. This scoring system is used to assess abnormalities in the cerebral WM, cortical GM, deep GM, and cerebellum. Cerebral WM abnormality was assessed using six items that were graded between 0 and 4: (1) cystic degeneration, (2) focal signal abnormalities, (3) delayed myelination, (4) corpus callosum thinning, (5) lateral ventricular dilatation, and (6) WM volume reduction. Cortical GM abnormality was assessed using three items that were graded between 0 and 4: (1) signal abnormality, (2) delayed gyration, and (3) extracerebral cerebrospinal fluid space dilatation. Deep GM and cerebellar abnormalities were assessed using two items that were graded between 0 and 4: (1) signal abnormality and (2) volume reduction. The total scores for each area were calculated separately, and each region was categorized as having no abnormality, mild abnormalities, moderate abnormalities, or severe abnormalities. A global brain abnormality score was calculated by summating the four regional total scores, and participants were classified as being normal or having mild, moderate, or severe brain injury based on this score.

All images were interpreted by a pediatric neuroradiologist who was blinded to all clinical data except gestational age and birth weight.

### Statistical analysis

Before performing the analysis, we checked the dataset for missing data. Data were analyzed using a statistical software package (Statistical Package for the Social Sciences, version 25.0, SPSS Inc., Chicago, IL, USA).

Data regarding maternal and infant variables were presented using descriptive statistics, including median, interquartile range (IQR), frequency, and percentage. Fisher’s exact test was used to determine the association between categorical variables. The Mann–Whitney U test was used for between-group comparisons of ordinal qualitative variables (gestational age, birth weight, and Apgar score). For between-group comparisons of continuous variables, the unpaired Student’s t-test was used for normally distributed data, and the Mann–Whitney U test was used for non-normally distributed data. The Kolmogorov–Smirnov test and a visual inspection of histograms were performed to evaluate the distribution of quantitative variables.

To analyze the association between early morphine exposure and outcomes, we first conducted a univariate relative risk analysis on the recorded variables (gestational age; birth weight; sex; 1-min Apgar score; 5-min Apgar score; necrotizing enterocolitis; patent ductus arteriosus; administration of inotropes, hydrocortisone, and midazolam; cumulative morphine dose; ventilator use; surfactant use, maternal hypertension; antenatal and postnatal steroid treatment; and mode of delivery, IVH, LOS) because we considered them to be potential confounders^35^. All factors with a P value of < 0.05 in the univariate analysis were included in the final multivariable regression model. Modified log-Poisson regression with generalized linear models and a robust variance estimator (Huber–White) was applied for univariate relative risk analysis and to the models to adjust the relative risk for poor global brain abnormality scores. Negative binomial regression analysis was conducted to determine the effect of cumulative morphine exposure on the global brain abnormality score. To ensure that the primary outcome is not affected by gestational age and birth weight, we performed 1:1 matching of a group of infants who had received morphine in the first week of life and another group of infants who had not received morphine.

All statistical tests were two-tailed, and P values of < 0.05 were considered statistically significant.

## Data Availability

The data sets analyzed during this study are available from the corresponding author on reasonable request.
